# Pennsylvania Hospital—Surgical Wards

**Published:** 1851-12

**Authors:** Addinell Hewson

**Affiliations:** Resident Surgeon; Pennsylvania Hospital


### Pennsylvania Hospital—*Surgical Wards.—Service of* Drs. Fox and Norris.

*Cases discharged since August 15th,* 1851.

| | Cured. | Relieved. | Died. | Total. |
|---|---|---|---|---|
| Abscess, - - - - | 2 | 0 | 0 | 2 |
| Anchylosis of elbow, - - - - | 0 | 1 | 0 | 1 |
| Burns, - - - - | 11 | 2 | 3 | 16 |
| Cancer of lip, - - - - | 2 | 1 | 0 | 3 |
| " tongue, - - - - | 0 | 1 | 0 | 1 |
| " penis, - - - - | 1* | 0 | 0 | 1 |
| Caries of sternum, - - - - | 0 | 1 | 0 | 1 |
| Concussion, - - - - | 0 | 1 | 0 | 1 |
| Conjunctivitis, - - - - | 0 | 1 | 0 | 1 |
| Contusion, - - - - | 16 | 2 | 0 | 18 |
| Disease of hip, - - - - | 0 | 1 | 0 | 1 |
| " testicle, - - - - | 0 | 1 | 0 | 1 |
| " tibia, - - - - | 0 | 1 | 0 | 1 |
| Dislocations 2, viz : | | | | |
| Humerus, - - - - | 1 | 0 | 0 | 1 |
| Femur, - - - - | 1† | 0 | 0 | 1 |
| Fistula in ano, - - - - | 1 | 1 | 0 | 2 |
| Fractures, simple, 40, viz : | | | | |
| Skull, - - - - | 0 | 0 | 2 | 2 |
| Inferior maxillary, - - - - | 1 | 0 | 0 | 1 |
| Clavicle, - - - - | 3 | 2 | 0 | 5 |
| Humerus, - - - - | 4 | 1 | 0 | 5 |

* By amputation.

† Reduced in ten minutes under the influence of ether, after three previous unsuccessful attempts without it, one of which lasted three hours.

| | Cured. | Relieved. | Died. | Total. |
|---|---|---|---|---|
| Condyles of humerus, - - - | 3 | 1 | 0 | 4 |
| Forearm, (both bones,) - - - | 0 | 2 | 0 | 2 |
| Radius, - - - - | 3 | 0 | 0 | 3 |
| Ulna, - - - - | 2 | 0 | 0 | 2 |
| Ribs, - - - - | 4 | 0 | 0 | 4 |
| Ilium, - - - - | 1 | 0 | 0 | 1 |
| Femur, - - - - | 2 | 1 | 0 | 3 |
| Patella, - - - - | 1 | 0 | 0 | 1 |
| Leg, (both bones,) - - - - | 6 | 0 | 0 | 6 |
| " ununited, - - - - | 1 | 0 | 0 | 1 |
| Fractures, compound, 21, viz: | | | | |
| Skull, - - - - | 0 | 0 | 1 | 1 |
| Face, - - - - | 1 | 0 | 0 | 1 |
| Nose, - - - - | 0 | 1 | 0 | 1 |
| Shoulder, - - - - | 0 | 0 | 1 | 1 |
| Forearm, - - - - | 2* | 0 | 0 | 2 |
| Fingers, - - - - | 9 | 0 | 0 | 9 |
| Thigh, - - - - | 0 | 0 | 1 | 1 |
| Leg, - - - - | 2 | 0 | 0 | 2 |
| Foot, - - - - | 3 | 0 | 0 | 3 |
| Gonorrhœa, - - - - | 7 | 1 | 0 | 8 |
| Hemorrhage from urethra, - - - | 0 | 1 | 0 | 1 |
| Hydrocele, - - - - | 1 | 3 | 0 | 4 |
| Inflammation of ear, - - - - | 0 | 1 | 0 | 1 |
| " knee, - - - - | 1 | 0 | 0 | 1 |
| " leg, - - - - | 2 | 1 | 0 | 3 |
| " foot, - - - - | 1 | 0 | 0 | 1 |
| Injury of wrist, - - - - | 0 | 1 | 0 | 1 |
| " knee, - - - - | 1 | 2 | 0 | 3 |
| Iritis - - - - | 2 | 1 | 0 | 3 |
| Onychia, - - - - | 6 | 0 | 0 | 6 |
| Ophthalmia, - - - - | 1 | 0 | 0 | 1 |
| Paronychia, - - - - | 15 | 1 | 0 | 16 |
| Scalds, - - - - | 3 | 0 | 0 | 3 |
| Sprains, - - - - | 3 | 0 | 0 | 3 |
| Syphilis, (primary,) - - - - | 8 | 3 | 0 | 11 |
| " (secondary,) - - - - | 5 | 3 | 1† | 9 |
| " (tertiary,) - - - - | 1 | 0 | 0 | 1 |
| Tumors, - - - - | 1 | 0 | 0 | 1 |
| " of eyelid, - - - - | 0 | 1 | 0 | 1 |
| " of breast, - - - - | 1 | 0 | 0 | 1 |
| Ulcers, simple, - - - - | 11 | 5 | 0 | 16 |
| " scrofulous, - - - - | 0 | 1 | 0 | 1 |
| " varicose, - - - - | 1 | 0 | 0 | 1 |
| " of breast, - - - - | 1 | 0 | 0 | 1 |
| Wounds, gunshot, - - - - | 3 | 2 | 2 | 7 |
| " incised, - - - - | 5 | 1 | 0 | 6 |
| " lacerated, - - - - | 6 | 2 | 1‡ | 9 |
| " penetrating, - - - - | 2 | 0 | 0 | 2 |
| | 172 | 52 | 12 | 236 |

* One by amputation.

† From hemorrhage from nasal artery.

‡ From mania à potu.

*Gunshot wounds of Forearm and Thorax.—Recovery.*—Noble McClintock, aged 18, was brought to the Hospital on the evening of the 17th of August, having been shot half an hour previous, whilst engaged in a firemen's riot. His countenance was pallid and expressive of great nervous depression. Pulse 70 and feeble. Skin moist.

On examination, there were found two wounds in the right forearm, and one in the right thorax, evidently produced by a small pistol ball, which had passed through the fleshy portion of the supinator longus, one inch below the bend of the elbow, and then entered the right thorax between the seventh and eighth ribs, a little posterior to a vertical line from the nipple. The wound in the thorax was round; lips inverted, blackened, and presented all the appearances of the ball having passed through the parietes of the thorax. There were no bloody sputa, or physical signs of the lung having sustained any injury; the vesicular murmur was equally audible on both sides, and percussion resonant. There was no tenderness of the abdomen, save at the scrobiculus cordis (where it was but slight.) There was no spasm of the diaphragm, nor were there any signs of the ball having passed round the thorax beneath the integuments, and making a wound of exit at an opposite point.

Having drawn the edges of the wounds together by adhesive strips, the cold water dressings were applied. Perfect rest enjoined; ordered absolute diet.

Reaction came on very gradually, but no hemorrhage occurred.

On the third day after the accident, some symptoms of pleuro-pneumonia of a local character manifested themselves, and were treated by dry cupping, mercury and diaphoretics. They subsided in a short time. Some small pieces of clothing were discharged from the wound, which afterwards gradually cicatrized, and the patient was discharged well on the 16th of September, thirty days after the occurrence of the accident.

He has since been several times to the Hospital and stated that his health continues good. On being questioned as to any previous illness, he stated that some years ago he had had an attack of pleurisy on the right side. Such having been the case, it is not difficult to account for the absence of any symptoms of collapse of the lung, by the adhesions which such an inflammation usually establishes between the two pleuræ.

The absence of bloody sputa still leaves a doubt as to the ball having entered the lung, but we have more than one case on record, from good authority, of wound of the lung without such symptoms manifesting themselves; and although we are not confident that the ball did enter the lung in this case, we have little doubt but that an autopsy would reveal it encysted somewhere within the parietes of the thorax.

One of the fatal cases of gunshot wound, occurred in an attempted burglary at a farm below the city. The burglar was shot from a second story window, with large buck-shot. Many of them entered the scalp, one or two producing indented fracture of the skull. Others entered the right thorax, wounding the lung. The man was brought to the Hospital about an hour after the accident, but in a dying state. He died two hours after his admission, never having reacted at all; and, on examination, the right pleura was found filled with blood—there was no injury done to the brain.

The other fatal case was the result of an accident which occurred on board of one of the steamers for Boston. When about to leave port, a small cannon was accidentally discharged at the moment when the mate was at its muzzle, attempting to alter its elevation. The whole charge, consisting of powder and wadding, passed obliquely between his thighs, causing a very extensive wound.

The chief part of the scrotum, the right testicle, except a portion of the epididymus, a portion of the left testicle, the integuments and cellular tissue of the perineum, the arms and lower portion of the rectum, and the tissues between the anus and inner side of the right thigh, together with a portion of the upper extremities of the muscles of that thigh, were carried away. The urethra and crura of the penis were laid bare, and the tuberosity of the ischium was exposed, though not denuded of its periosteum.

The hæmorrhage was very profuse, and although a number of vessels were tied immediately on his reaching the Hospital, it was renewed when reaction was established—more vessels were then tied, and the whole surface of the wound sprinkled with powdered matico. Even with this it was impossible to entirely arrest the oozing, and the patient lost more than a pint of blood in the course of the following night.

The sufferer had been a stout healthy young man, aged 26, and resisted for a long time the depressing influence of such an injury. The sloughs following the wound were deep, and the suppuration profuse but it was not until the 22d day after the accident, that he died from exhaustion.

ADDINELL HEWSON, Resident Surgeon.

*Pennsylvania Hospital, Nov. 15th*, 1851.

